# Supplementary material for: EPSP Synthase-Depleted Cells Are Aromatic Amino Acid Auxotrophs in Mycobacterium smegmatis
Source: Microbiol Spectr. 2021 Dec 22;9(3):e00009-21. doi: 10.1128/Spectrum.00009-21 (PMC8694188; doi:10.1128/Spectrum.00009-21)
Supplement: SUPPLEMENTAL FILE 1 — Supplemental material. Download SPECTRUM00009-21_Supp_1_seq2.pdf, PDF file, 1.0 MB [file spectrum00009-21_supp_1_seq2.pdf]

Supplemental Material

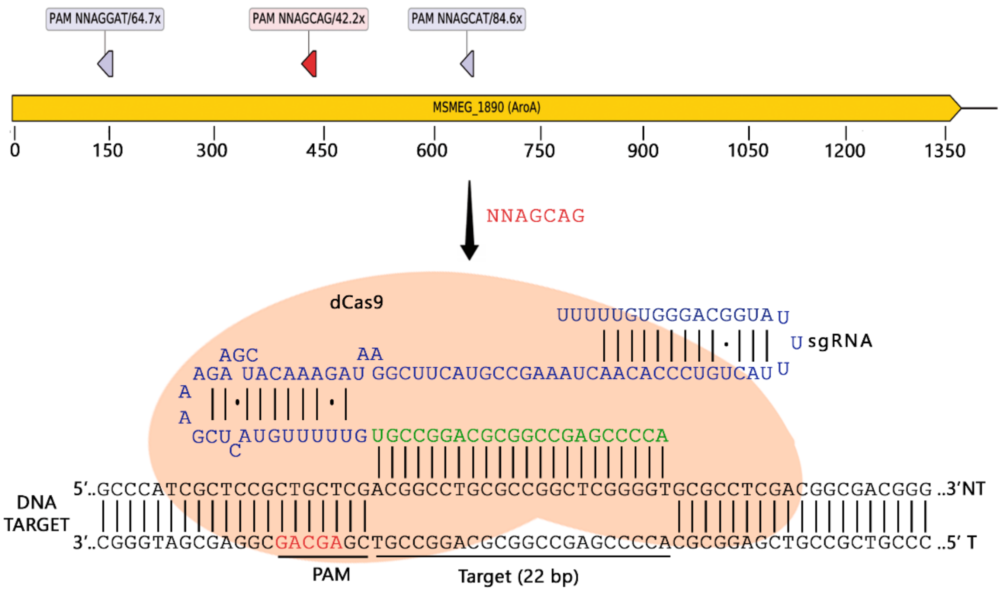

Figure S1. Knockdown of EPSPS-encoding gene *aroA* in *M. smegmatis* using CRISPR interference (CRISPRi). Upper part: Location of PAM sequences inside *aroA* locus used in this study. From left to right: 5'- NNAGGAT-3', 5'-NNAGCAG-3' and 5'- NNAGCAT-3'. The repression strength of each PAM sequence, according to Rock et al. (2017) (1), is also depicted. **Lower part**: Schematic representation of CRISPRi system associated with *aroA* locus at a target region adjacent to PAM "5'-NNAGCAG-3'". Dead Cas9 (dCas9) is represented in peach color, sgRNA as a single RNA chain in blue with annealing portion in green and paired with the non-template (NT) strand of target DNA. The "5'-AGCAG-3'" from PAM is depicted in red in the template (T) strand of target DNA in 3'-5' orientation.

MESG

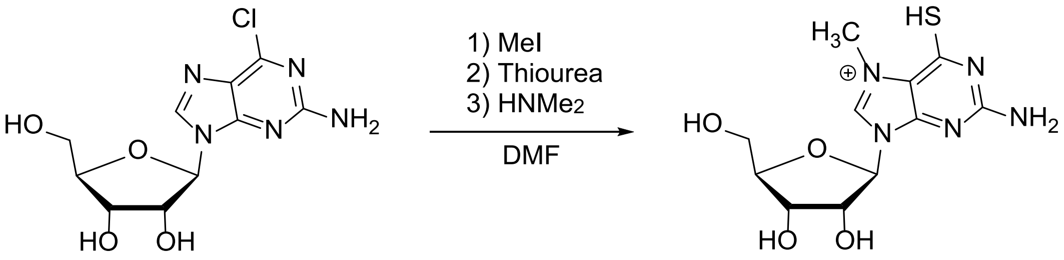

6-Chloroguanine riboside **MESG**

Figure S2. MESG synthesis. In a two-neck round bottom flask, under argon atmosphere, 6-chloroguanine riboside (4.00 g, 13.25 mmol) was dissolved in dry dimethylformamide (10 mL). Then, methyl iodide (4 mL, 64.25 mmol) was added and the mixture was stirred overnight at 30 °C. Excess methyl iodide was removed under vacuum together with part of DMF. Then, to the residual mixture, thiourea (2.00 g, 26.27 mmol) was added under an argon atmosphere and the mixture was stirred for an additional hour. Afterwards, the solution was neutralized with pure dimethylamine slowly added dropwise. The mixture was directly poured into stirred acetone (500 mL) to give a yellow precipitate which was further chromatographed on silica and eluted with ethyl acetate/1-propanol/water (5:2:1; v/v) yielding 0.620g (30%) of MESG. The compound was dried to a yellow solid and stored desiccated at -80 °C. ^{13}C NMR (D_2O) δ (ppm): 174.2, 156.0, 146.6, 141.1, 119.6, 90.8, 86.2, 75.1, 70.1, 61.3, and 35.4. HRMS (ESI): calc. for $[C_{11}H_{16}N_5O_4S+H]^+$: 314.0918; obt: 314.0916.

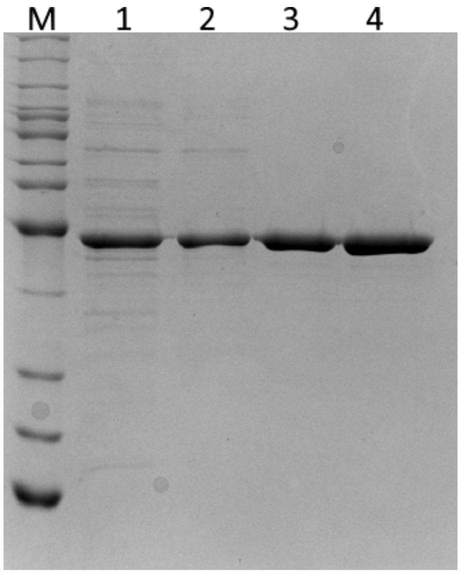

Figure S3. SDS-PAGE of samples from the purification steps of WT *Ms*EPSPS. Lane M: BenchMark Protein Leadder (Invitrogen). Lane 1: crude extract from soluble fraction of cell disruption. Lane 2: soluble fraction from the first column (Q-Sepharose Fast Flow). Lane 3: soluble fraction from the second column (Phenyl Sepharose HP). Lane 4: soluble fraction from the third column (Mono Q HR 16/10).

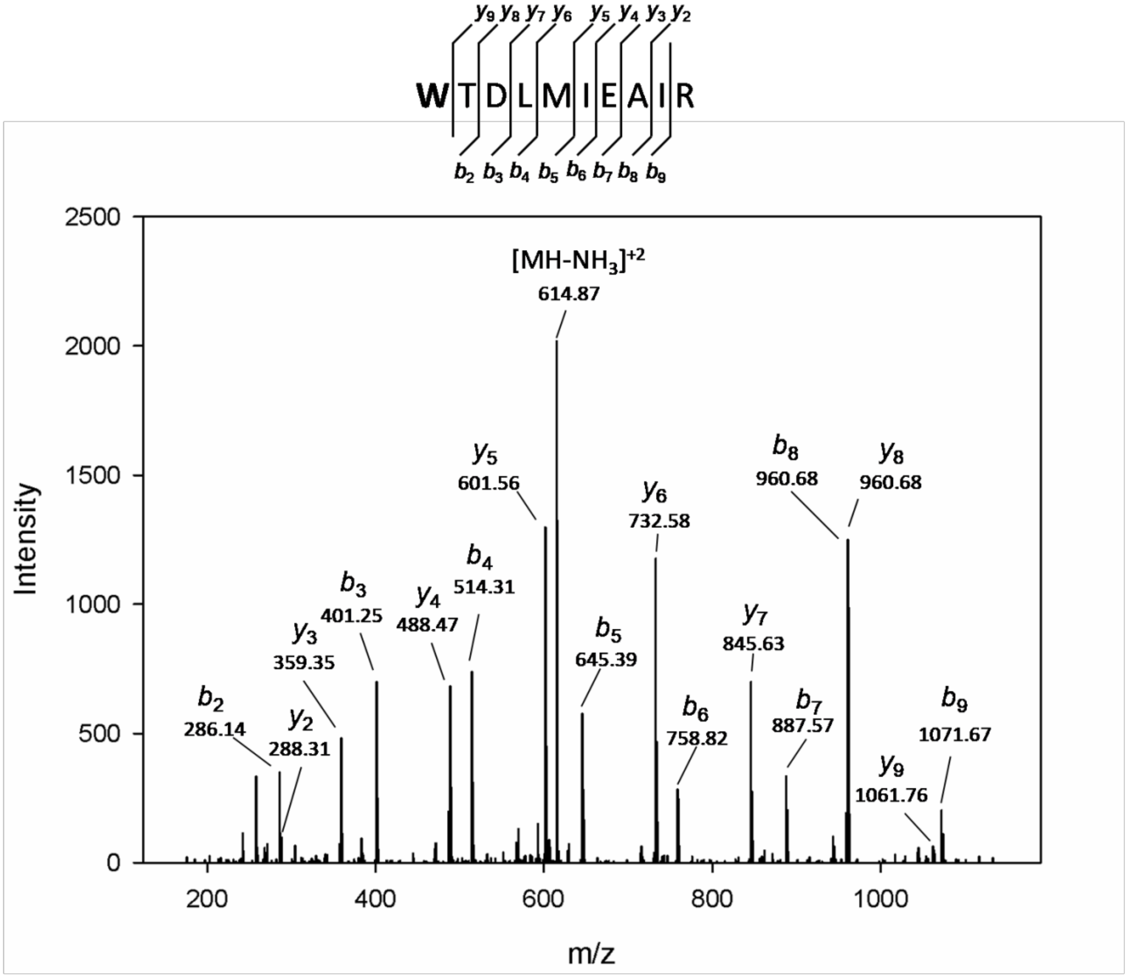

Figure S4. Representative spectrum of peptide containing the D61W mutation obtained by LC-MS/MS of *Ms*EPSPS D61W protein. Peptide sequence: **W**TDLMIEAIR. Point mutation marked in **bold**. Fragment b- and y-ions and their neutral loses are indicated.

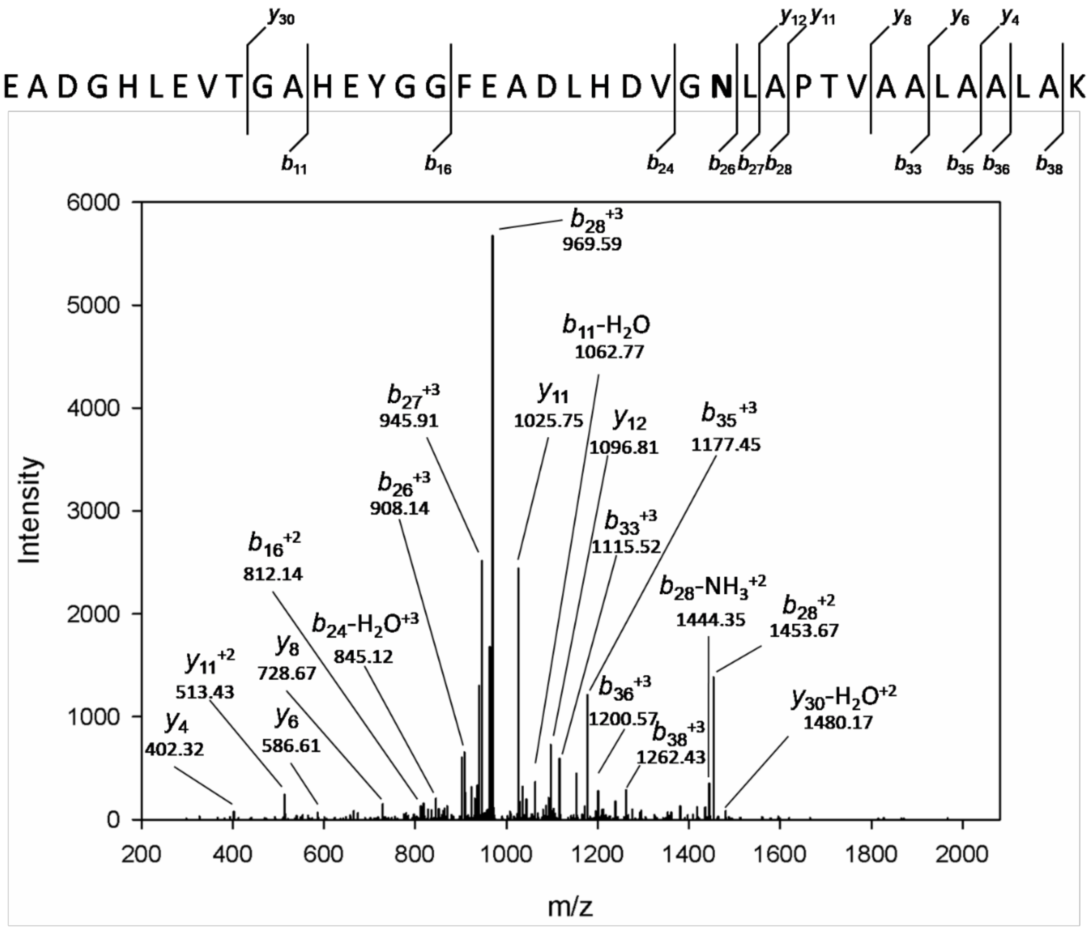

Figure S5. Representative spectrum of peptide containing the E321N mutation obtained by LC-MS/MS of *Ms*EPSPS E321N protein. Peptide sequence: EADGHLEVTGAHEYGGFEADLHDVG **N**LAPTVAALAALAK. Point mutation marked in **bold**. Fragment b- and y-ions and their neutral loses are indicated.

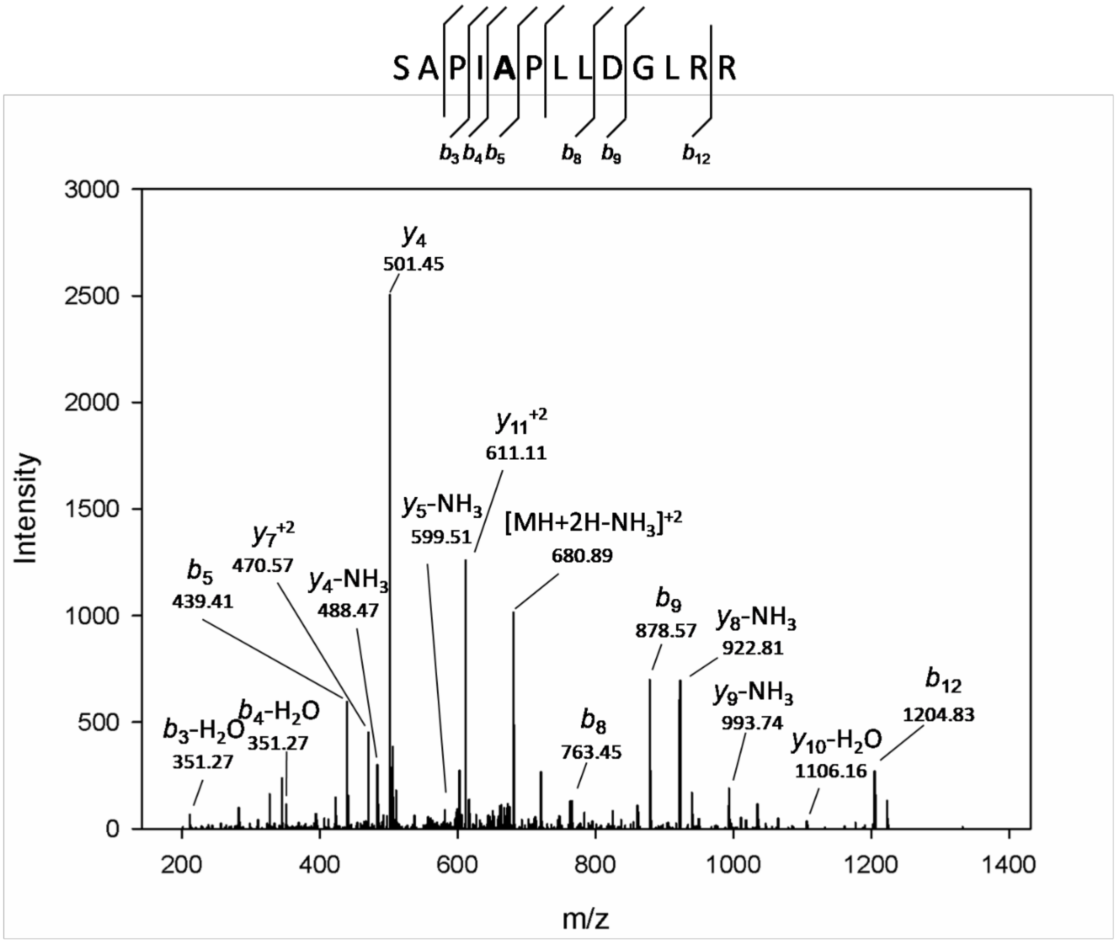

Figure S6. Representative spectrum of peptide containing the R134A mutation obtained by LC-MS/MS of *Ms*EPSPS R134A protein. Peptide sequence: S**A**PIAPLLDGLRR. Point mutation marked in **bold**. Fragment b- and y-ions and their neutral loses are indicated.

Table S1. Fixed and varying concentrations of substrates used in kinetic assays of *Ms*EPSPS.

EPSPS	Substrate	Fixed-saturating (μM)	Substrate	Varying range (μM)
**WT**	S3P	800	PEP	25 - 900
**D61W**	S3P	600	PEP	25 - 1200
**E321N**	S3P	600	PEP	25 - 10000
**R134A**	S3P	600	PEP	300 - 2900

Supplementary Results

MESG synthesis. High-resolution mass spectra (HRMS) were obtained on an LTQ Orbitrap Discovery mass spectrometer (Thermo Fisher Scientific). This system combines an LTQ XL linear ion-trap mass spectrometer and an Orbitrap mass analyzer. The analyses were performed through the direct infusion of the sample in MeOH/H_2O (1:1) with 0.1% formic acid (flow rate 10 mL/min) in a positive-ion mode using electrospray ionization (ESI). For elemental composition, calculations used the specific tool included in the Qual Browser module of Xcalibur (Thermo Fisher Scientific, release 2.0.7) software. ^{13}C NMR spectra were acquired on an Avance III HD Bruker spectrometer (Pontifical Catholic University of Rio Grande do Sul); chemical shifts (δ) were expressed in parts per million (ppm) relative to TMS (tetramethylsilane) used as an internal standard.

References

[1] Rock JM, Hopkins FF, Chavez A, Diallo M, Chase MR, Gerrick ER, Fortune SM. 2017. Programmable transcriptional repression in mycobacteria using an orthogonal CRISPR interference platform. Nature Microbiology, 2: 1–9. https://doi.org/10.1038/nmicrobiol.2016.274
